# Shifting Issues of Support Exchange Under 20-Year Implementation of Japanese Long-Term Care Insurance Program

**DOI:** 10.1093/geroni/igab046.1623

**Published:** 2021-12-17

**Authors:** Tomoko Wakui

## Abstract

Japan has faced numerous issues in the last twenty years with its mandatory long-term care (LTC) insurance program. This LTC insurance program obviously affected older adults’ informal support exchanges, reducing support from family and the community, which became more valuable, subjectively. Furthermore, changes in support have impacted older adults’ subjective well-being and children’s perceived care motivation. Additionally, a mandatory uniform system challenges the issue of tolerance of diversity, meaning how non-traditional families’ opinions be involved LTC situations. This symposium discusses unexpected shifting issues in Japan in the implementation of a public LTC program with a focus on older adults’ support exchanges. The first paper examines the long-term impacts of formal and informal support by examining the effects of implementing formal services. The second paper assesses a community’s role in relation to family in the presence of a public LTC program. The third paper examines the subjective impacts of older parents, who provided support to adult children and their reciprocal expectations of receiving LTC. The fourth paper, on the other hand, articulates reciprocal impacts on sons’ care motivation, which has become more important, since the introduction of the LTC program reinforced men’s participation in LTC. Finally, the fifth paper clarifies how a public uniform program accommodates informal support from non-traditional families when the program premises the presence of family in advanced care planning. Our findings have long-term implications for aging societies in relation to formal and informal support exchanges.

